# Aromatherapy for the treatment of PONV in children: a pilot RCT

**DOI:** 10.1186/s12906-016-1441-1

**Published:** 2016-11-09

**Authors:** Mathew B. Kiberd, Suzanne K. Clarke, Jill Chorney, Brandon d’Eon, Stuart Wright

**Affiliations:** 1 Department of Anesthesia, Pain Management and Perioperative Medicine, Dalhousie University, 1276 South Park Street, 231-C 10 West Victoria Building, Halifax, NS B3H 2Y9 Canada; 2 Department of Psychology and Neuroscience, Dalhousie University, Halifax, NS Canada; 3 Center for Pediatric Pain Research IWK Health Centre, Halifax, NS Canada; 4 Department of Pediatric Anesthesia, IWK Health Sciences Center, IWK Pediatric Anesthesia and Complex Pain Team, 5850/5989 University Ave, Halifax, NS B3K 6R8 Canada

**Keywords:** MeSH postoperative nausea and vomiting, Aromatherapy, Ambulatory surgical procedures, Pediatrics, Nausea, Antiemetics, Complementary therapies

## Abstract

**Background:**

Postoperative nausea and vomiting (PONV) is one of the most common postoperative complications of general anesthesia in pediatrics. Aromatherapy has been shown to be effective in treating PONV in adults. Given the encouraging results of the adult studies, we planned to determine feasibility of doing a large-scale study in the pediatric population.

**Methods:**

Our group conducted a pilot randomized controlled trial examining the effect of aromatherapy on post-operative nausea and vomiting in patients 4–16 undergoing ambulatory surgery at a single center. Nausea was defined as a score of 4/10 on the Baxter Retching Faces Scale (BARF scale). A clinically significant reduction was defined as a two-point reduction in Nausea. Post operatively children were administered the BARF scale in 15 min internals until discharge home or until nausea score of 4/10 or greater. Children with nausea were randomized to saline placebo group or aromatherapy QueaseEase™ (Soothing Scents, Inc, Enterprise, AL: blend of ginger, lavender, mint and spearmint). Nausea scores were recorded post intervention.

**Results:**

A total of 162 subjects were screened for inclusion in the study. Randomization occurred in 41 subjects of which 39 were included in the final analysis. For the primary outcome, 14/18 (78 %) of controls reached primary outcome compared to 19/21 (90 %) in the aromatherapy group (*p* = 0.39, Eta 0.175). Other outcomes included use of antiemetic in PACU (control 44 %, aromatherapy 52 % *P* = 0.75, Eta 0.08), emesis (Control 11 %, 9 % aromatherapy, *P* = 0.87, Eta = 0.03). There was a statistically significant difference in whether subjects continued to use the intervention (control 28 %, aromatherapy 66 %, *p*-value 0.048, Eta 0.33).

**Conclusion:**

Aromatherapy had a small non-significant effect size in treating postoperative nausea and vomiting compared with control. A large-scale randomized control trial would not be feasible at our institution and would be of doubtful utility.

**Trial registration:**

ClinicalTrials.gov NCT02663154.

## Background

Postoperative nausea and vomiting (PONV) is one of the most common postoperative complications of general anesthesia in pediatrics. Pediatric rates of nausea and vomiting are approximately double those of adult patients (approximately 40 %) [1, 2]. PONV is an unpleasant experience with potential secondary complications such as wound dehiscence, electrolyte abnormalities and aspiration pneumonia. PONV can also result in significantly delayed post anesthetic care unit (PACU) stays, which can lead to delayed hospital discharge [3]. In a study using a ‘willingness-to-pay technique,’ parents were willing to pay up to $80 to prevent nausea, suggesting that parents feel PONV is a significant problem [4].

Aromatherapy has been shown to be effective in treating PONV in adults [5–7]. Aromatherapy is the use of essential oils to alleviate emotional or physical discomfort. The cellular and physiological means by which aromatherapy acts is poorly understood [8]. Meta-analysis of four previous studies (215 subjects) failed to show a significant effect of isopropyl alcohol compared with standard treatment for relief of nausea. [8] Peppermint was also examined in this Cochrane review and there was insufficient quality evidence to show an effect of peppermint-based aromatherapy. However, this review did not include the largest trial in adult aromatherapy for PONV. In 2013 Hunt et al. enrolled 1151 adults in a four armed randomized control trial for aromatherapy and showed that the blend (ginger, peppermint, spearmint and cardamom) reduced nausea compared to saline: 82.4 % reported reduced nausea with blend compared to control 39.7 % (*P* < 0.001) [6]. A number of smaller studies have also shown efficacy of aromatherapy in treating adult PONV [5, 7, 9].

QueaseEase™ (Soothing Scents, Inc, Enterprise, AL) is one of the proprietary blends of aromatherapy that has been shown to be effective in treating PONV in adults. In a study of 339 patients, 94 reported nausea and were randomized to aromatherapy or placebo. The intervention resulted in a reduction in mean visual analogue nausea score of 5.4 pre aromatherapy to 3.4 post aromatherapy *p* = 0.01. This difference was significantly greater than nausea reduction by placebo: 5.6 pre and 4.4 post *P* = 0.03 [5]. Given the encouraging results of the first adequately powered adult study, we planned to determine feasibility of doing a large-scale study in the pediatric population.

Herein we report a pilot randomized control trial to assess the feasibility of a larger scale randomized clinical trial. The aim is to identify refinements necessary to the study protocol, assess costs, and obtain a valid estimate for power calculations. Hence, to answer in future studies whether aromatherapy is an effective therapy for the management of PONV in the pediatric population.

## Methods

The study was conducted at the Isaac Walton Killam (IWK) Health Centre in Halifax, Nova Scotia. After institutional ethics review, participants were recruited in two discrete collection periods by three research assistants (July 14-Sept 23 2014 and March 24-May 19 2015). Inclusion criteria included children aged 4–16, Anesthesia Society of America Physical Status I or II (ASA I or II), absence of neurodevelopmental disorders, and undergoing elective day surgery. Exclusion criteria included patient or family refusal, allergy or sensitivity to aromatherapy components, inability to smell or failure to meet the inclusion criteria. Of note, the protocol was modified at the study mid-point to increase enrollment. Originally, only otolaryngology and ophthalmology patients were included, but this was expanded to the previously described inclusion criteria due to low rates of PONV.

### Study intervention

**Fig. 1:**
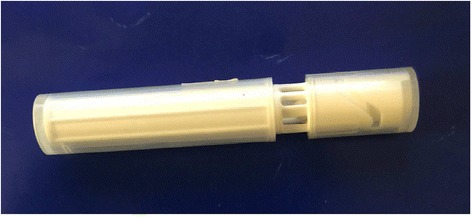
An example of a placebo or aromatherapy inhaler is shown above

The intervention was a proprietary blend of aromatherapy essential oils (QueaseEASE™). The mixture included (*Lavandula angustifolia*, *Mentha Spicata*, *Mentha xpiperita* and *Zingiber officinale*) in equal proportions and is registered with Health Canada (Natural Product Number 80036451). The control was with identical housing but contained only saline (Fig. 1).

### Measurement tool

#### BARF scale

The Baxter Retching Faces (BARF) scale is a pictorial nausea scale of 0–10 with 6 faces. This tool was validated in the paediatric emergency room, with paediatric cancer inpatients and in the paediatric postoperative care unit [10]. This instrument has achieved construct validity for assessment of severity of paediatric nausea. The scale also has convergent and discriminatory validity and can detect changes after therapy. Originally validated for patients aged 7–18 years old, this scale has been used in ages as low as 4 [10].

### Procedure

A pilot trial designed to accurately replicate future larger study design was undertaken as follows.

On the day of their surgical procedure, children were screened using the inclusion and exclusion criteria. Children whom met the criteria were enrolled after obtaining informed consent from the parent with assent from the child. Enrolment took place prior to the start of the scheduled procedure.

Upon enrollment, charts were reviewed for demographic data (age, sex, weight), co-morbidities, previous anesthetics, and previous PONV. Attending anesthesiologists were not made aware of the subject’s participation in the study and there were no attempts made to alter standard anesthetic management. Not standardizing anesthesia management improves external validity and applicability to the general pediatric peri-operative population.

Post-operatively subjects went to the first stage post anesthetic care unit (PACU 1) until they met discharge criteria. They were then moved to PACU 2 (post recovery) until discharge home. Upon arrival to PACU 1, the research assistant assessed for nausea using the BARF scale at 15-min intervals or if the patient or nurse reported nausea. The nurse also independently assessed nausea at 15-min intervals. If the patient reported a BARF scale of 4 or greater they were randomized to the intervention aromatherapy or a saline inhaler. Randomization was by block 6 design. Concealment was maintained by using sequentially numbered opaque envelopes containing the identical appearing intervention and control inhalers (Fig. 1). Upon the report of nausea, the patient was given the inhaler with instructions on its use. Specifically, subjects were asked to take deep regular breaths through their nose with the inhaler in front of their nose. They were told to do so as long as they wanted and to repeat as necessary until the nausea passed. Based on the preference of the participant, the inhaler was held by either the participant, a parent, or the research assistant. BARF scale was re-evaluated after sufficient exposure (defined as 5 respiratory cycles of nasal breathing using the inhaler within 30 cm from their nares). The primary outcome was defined as a 2-point reduction in the BARF-Scale. A two-point difference is a one-face difference on the pictorial scale. This difference would be analogous to a significant clinical effect in the pain literature and has been used in the pediatric nausea literature [10]. Additional BARF scale measurements were collected at 15-min intervals until the time of discharge.

### Statistical analysis

#### Power consideration

The nature of this study was to select a convenience sample of 40 patients (20 in each group) to serve as an estimate for power in a future study. The current literature does not contain a study similar enough that assumptions for a sample size calculation would be valid. A sample size of 40 patients is generally sufficient to create an accurate estimate of variance in which a future power calculation could be made.

#### Analysis

SPSS version 23.0 was used for analysis. Descriptive statistics were generated and are presented as means with standard deviation or as percentages. T-tests and chi-square were used to compare groups on baseline data including child age, surgical procedure, previous PONV and co-morbidities. T-tests (or Analysis of Covariance) were used to examine differences on primary.

## Results

**Fig. 2:**
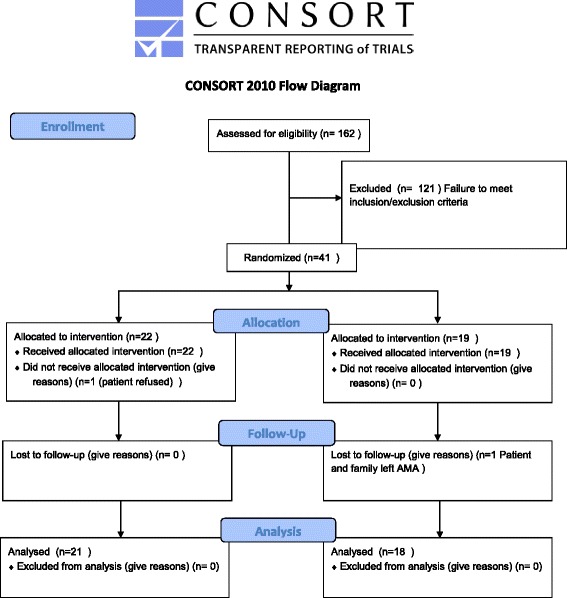
Consort Flow Diagram

A total of 162 subjects were screened for inclusion in the study. Of these subjects, 4 surgeries were cancelled, 2 were changed to same day admission, 2 parents withdrew consent, and 2 patients were excluded retrospectively for ASA status III. The overall rate of nausea as defined as 4/10 on the BARF nausea scale was 41/152 (27 %). Randomization occurred in 41 subjects of which 2 were excluded post randomization (1 subject in each arm [1], for failure to meet exposure criteria and [1] for leaving before assessment (Fig. 2)).

Age, sex, ASA status, total time in operating room, PONV prophylaxis and pain management were not significantly different between intervention and control groups (Table 1). There were differences in the type of surgical procedures between groups. The major difference was a greater portion of patients in the aromatherapy group had ENT procedures compared with the control group (81 % versus 44 %) (Table 1). When comparing the groups for nausea prophylaxis, 78 % of the control and 71 % of the aromatherapy group received Ondansetron and 94 % of control and 86 % of the aromatherapy group received dexamethasone. The groups were also compared for multimodal analgesia, including acetaminophen: 94 % of controls and 95 % of aromatherapy subjects received multimodal analgesia.

**Table 1:** Summary of demographic data between control and aromatherapy arm

	Control	Aromatherapy
Age (YEAR)	8.5 (95 % CI ±3.4)	6.9 (95 % CI ±3.1)
Sex (%male)	9/18 (50 %)	12/21 (57.1 %)
ASA (I or II)	100 %	100 %
Surgery		
ENT	8/18 44 %	17/21 81 %
Ophthalmology	5/18 27.8 %	1/21 4.8 %
Plastic surgery	2/18 11.1 %	0/21 0 %
Orthopaedics	0/18 0 %	0/21 0 %
Dental	1/18 5.6 %	3/21 14.3 %
Urology	0/18 0 %	0/21 0 %
General surgery	2/18 11.1 %	0/21 0 %
OR time (minutes)	66 (95 % CI ±18)	64 (95 % CI ±34)
PONV Prophylaxis		
TIVA	4/18 22 %	4/21 19 %
Ondansetron	14/18 77.8 %	15/21 71 %
Dexamethasone	17/18 94.4 %	18/21 85.7 %
Pain management		
Acetaminophen	17/18 94.4 %	20/21 95.2 %
Ketorolac	6/18 33 %	2/21 9.5 %
Morphine dose (mg/kg)	0.14 (95 % CI ±0.1)	0.13 (95 % CI ±0.09)

Continuous data were expressed as mean and 95 % CI. Otherwise data expressed as percentage of subjects meeting outcome

For the primary outcome, 14/18 (78 %) of controls reached the cutoff of a 2-point reduction on the BARF scale compared to 19/21 (90 %) in the aromatherapy group (*p* = 0.39, Eta 0.175) (Table 2). Other outcomes included use of antiemetic in PACU (control 44 %, aromatherapy 52 % *P* = 0.75, Eta 0.08), emesis (Control 11 %, 9 % aromatherapy, *P* = 0.87, Eta = 0.03) and reduction in BARF scale magnitude at 15-min post intervention [control 3.8 (95 % CI ±3.7), aromatherapy 3.2 (95 % CI ±2.4), *p*-value = 0.52, Eta = 0.01)] (Table 2). There was a statistically significant difference in whether subjects continued to use the intervention (control 28 %, aromatherapy 66 %, *p*-value 0.048, Eta 0.33) (Table 2). Subjects were considered to have continued the intervention if they used the intervention beyond the first 15 min.

**Table 2:** Summary of primary and secondary outcomes

	Control	Aromatherapy	*p*-value	Effect (Eta)
2 point reduction on BARF scale (Y/N)	14/18 (77.8 %)	19/21 (90.5 %)	0.39	0.175
Use of pharmacological rescue (Y/N)	8/18 (44.4 %)	11/21 (52.4)	0.75	0.079
Emesis (Y/N)	2/18 (11.1 %)	2/21 (9.5 %)	0.87	0.026
Continued use of intervention (Y/N)	5/18 (28 %)	14/21 (66 %)	0.048	0.33
Reduction in BARF scale magnitude	3.8 (95 % CI ±3.7)	3.2 (95 % CI ±2.4)	0.52	0.01

Reduction in BARF magnitude is expressed as mean and 95 % CI. The means were compared using T-Test with calculation of effect size. Otherwise data are expressed as percentage of subjects meeting the outcome (binary Y/N) and means were compared using Chi-Square test with calculation of effect size

### Study feasibility indicators

There were 46 study days over a period of 6 months resulting in 3.2 subjects recruited per study day and a randomization rate of 0.9 subjects per day. Approximate cost of the study was $8 000 mainly from research assistant salary and to a small extent, materials. Thus, a cost of $205 for each randomized subject.

## Discussion

The primary goal of the current study was to assess the feasibility of a larger trial of aromatherapy for PONV in children. The primary outcome of 2-point reduction in the BARF scale was a surrogate for moderate PONV. The outcome had only a small-modest effect size (control 78 %, aromatherapy 90 %, Eta = 0.175). In order to be powered to a significance level (α) of 0.05 and power of 0.8, the sample size would need to be approximately 514 per group (making no adjustments for attrition) using the effect size of the primary outcome. Alternatively using the incidence of nausea the sample size would be 190 patients per group. Given the recruitment rates of the study the lowest estimate of number children we would need to screen is 1 200. Concrete, less subjective outcomes such as pharmacological intervention or emesis would require even larger sample sizes to find any difference as evidenced by the low effect sizes found in this study (0.08 and 0.03 respectively).

Nausea rates at our center (27 %) were close, though somewhat lower, to literature values [2, 6]. Compliance with recommendations for PONV prophylaxis are consistent with recommendations from major society guidelines [1]. Most children in this study received both Ondansetron (74 %) and dexamethasone (95 %) as intraoperative prophylaxis. Multimodal analgesia was utilized in most patients to reduce narcotic use (95 %). The widespread use of multi-modal analgesia and PONV prophylaxis in our center likely has resulted in our lower rates of nausea.

Limitations of the study include low planned sample size, as this was a pilot study. Unreliability of the outcome measurement (BARF scale) in the youngest children may also contribute to error. Although the BARF scale has been validated down to 4 years old, there is variability in children’s ability to self-report on internal experiences in this age group that may have influenced their use of this scale [11].

Despite randomization there was a difference in the types of surgeries patients in each group received. For example, more patients in the control group had Ophthalmological surgery compared with aromatherapy (28 % versus 5 %). This was likely balanced by a higher portion of aromatherapy patient’s having ENT surgery. Initial nausea scores were not different between the two groups. In future study stratification by surgery may be necessary. Furthermore, the primary outcome measurement is an indirect surrogate rather than a concrete outcome. As PONV can occur for a period spanning longer than the time patients spend in day surgery, this study would not account for any PONV experienced, and possibly prevented by the intervention, *en* route home or over the first 24 h. Modifiable limitations include better concealment. Despite a delivery system with controlled exposure to the therapy (twist top) the aroma rapidly penetrated the area around the patient. Researchers and nurses correctly identified intervention versus control in all cases. In future studies nose plugs could be considered.

Future directions may include an adequately powered study with more attention to concealment and the inclusion of less subjective outcomes (reduction in emesis, and reduction in rescue anti-nausea medications). However, given the small effect size, the value is dubious. Other areas to consider exploring are the effects of aromatherapy on PONV, comfort, anxiety and pain in the extended post-operative period (i.e., over the following 24–48 h). Subjects in the aromatherapy group were more likely to continue using the intervention compared to the control group (66 % versus 28 % *p*-value 0.048, Eta 0.33). This may suggest that the aromatherapy could be having another unmeasured effect on patient comfort. This effect may be a reduction in anxiety. In future studies this should be explored further (measures of pain, comfort or anxiety reduction for example).

## Conclusion

This pilot study revealed methodological flaws that need to be corrected before embarking on a larger randomized control study. A larger trial would likely need to take place at a larger center or be multi-center in order to speed recruitment.

## Abbreviations

ASA: Anesthesia Society of America
BARF: Baxter Retching Faces
ENT: Ears nose throat
FPS-R: Faces of pain scale revised
IWK: Isaac Walter Killam
PACU: Post Anesthetic Care Unit
PONV: Postoperative nausea and vomiting
VAS-N: Visual Analogue Scale – Nausea
